# Expression of PD-1 and PD-L1 increase in consecutive biopsies in patients with classical Hodgkin lymphoma

**DOI:** 10.1371/journal.pone.0204870

**Published:** 2018-09-27

**Authors:** Peter Hollander, Rose-Marie Amini, Beatrice Ginman, Daniel Molin, Gunilla Enblad, Ingrid Glimelius

**Affiliations:** 1 Clinical and Experimental Pathology, Department of Immunology, Genetics and Pathology; Uppsala University, Uppsala, Sweden; 2 Experimental and Clinical Oncology, Department of Immunology, Genetics and Pathology; Uppsala University, Uppsala, Sweden; European Institute of Oncology, ITALY

## Abstract

High expression of programmed death receptor 1 (PD-1) and its ligand (PD-L1) by leukocytes in primary classical Hodgkin lymphoma (cHL) is associated with inferior outcome. However, it is unclear how expression varies during disease progression, and in the event of relapse. Our aim was to study PD-1 and PD-L1 in consecutive biopsies from *untreated* and *treated* cHL patients. We screened pathology registries from 3500 cHL patients. Eleven patients had a diagnostic cHL biopsy and a previous benign lymph node biopsy reclassified as cHL when reviewed and designated as the *untreated*. Thirty patients had a primary and a relapse biopsy, designated as the *treated*. Biopsies were immunostained to detect PD-1+ and PD-L1+ leukocytes, and PD-L1+ tumor cells. In the *untreated*, none of the markers were statistically significantly different when biopsies 1 and 2 were compared. In the *treated*, 19, 22, and 18 of 30 cases had increased proportions of PD-1+ leukocytes, PD-L1+ leukocytes and PD-L1+ tumor cells, respectively, and were all statistically significantly increased when primary and relapse biopsies were compared. PD-1 and PD-L1 most likely increase due to primary treatment with chemotherapy and radiotherapy, which could have implications regarding treatment with PD-1 inhibitors.

## Introduction

Classical Hodgkin lymphoma (cHL) has an overall good prognosis, but a proportion of patients suffer from refractory disease or relapse, with an increased risk of early death[1]. Programmed death receptor 1 (PD-1) and its ligand (PD-L1) are expressed to variable degrees in inflammatory conditions[2, 3] and malignancies[4–6]. Checkpoint inhibitors targeting the PD-1 pathway are effective treatment options in relapsed and refractory cHL[7, 8], but in first line the efficacy of these drugs is unknown. Consequently, the biological dynamicity of the PD-1 pathway in cHL during disease progression needs to be described. Patients with primary cHL with high proportions of both PD-1+ and PD-L1+ leukocytes have inferior outcome[9], but nothing has been published about how expression of PD-1 and PD-L1 evolves over time and when primary and relapse biopsies are compared.

cHL is characterized by a tumor microenvironment that consists of a variable infiltration of different leukocytes and few malignant Hodgkin and Reed-Sternberg (HRS) cells[10, 11]. This is a defining feature[12], which distinguishes cHL from other malignancies. Only a few studies have investigated how PD-1 and PD-L1 changes over time in other *untreated* malignant tumors. Higher proportions of PD-1+ macrophages in colorectal carcinoma have been associated with a longer disease duration[13]. In a previous study from our research group, there was a tendency for patients with cHL with high proportions of PD-L1+ leukocytes to present with advanced tumor stage[9]. It has never been studied whether PD-1 and PD-L1 are heterogeneously expressed in paired biopsies from patients with *untreated* cHL.

The PD-1 pathway may also become upregulated due to longer disease duration and following anti-cancer treatment in relapsed malignancies[13, 14]. Increased proportions of PD-L1+ tumor cells in non-small cell lung cancer (NSCLC)[15], and PD-1+ lymphocytes in multiple myeloma[16] were observed at relapse compared to primary diagnosis. It is unclear to what extent the PD-1 pathway changes in cHL when primary and relapse samples are compared.

Our aim was to determine how expression of PD-1 and PD-L1 develops both during disease progression in a unique material of *untreated* patients, and in primary *treated* patients that relapsed. Additionally, we wanted to explore whether expression of PD-1 and PD-L1 in relapse samples was related to survival after relapse. Our hypothesis was that higher proportions of PD-1+ and PD-L1+ leukocytes and PD-L1+ HRS cells would be associated with longer disease duration and relapse.

## Materials and methods

### Patients and tissue samples

**Fig 1:**
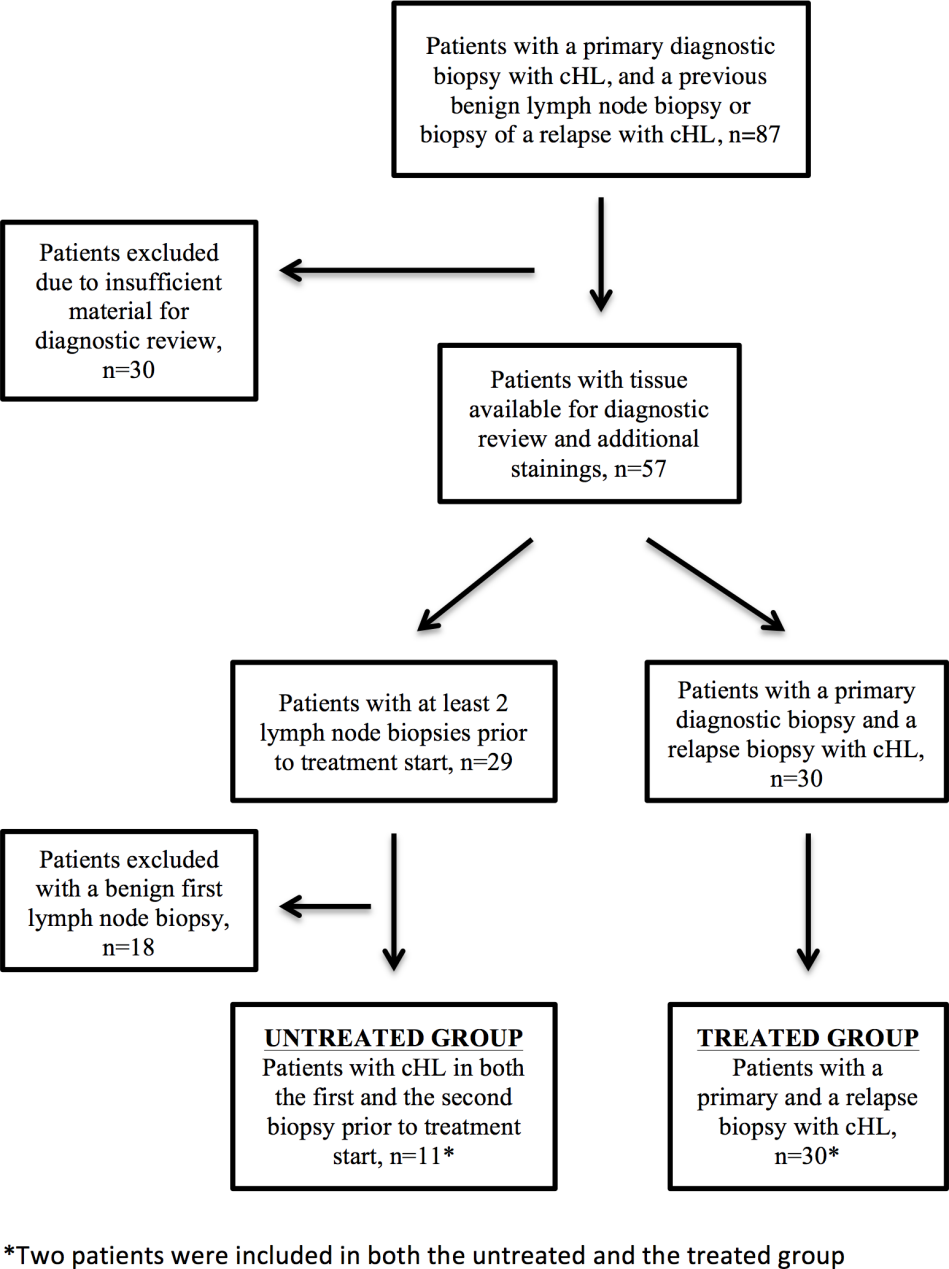
Flowchart of patients included in the study for the *untreated* and the *treated* group.

Pathology records during the years 1980–2015 in three Swedish referral pathology departments (Uppsala, Umeå and Lund) were screened for all cHL cases (n = 3500). Among them, 87 patients diagnosed with primary cHL that had at least one lymph node biopsy taken prior to the cHL diagnostic date were identified. Sections and formalin-fixed, paraffin embedded (FFPE) tissue blocks from these patients were collected and cases with remaining tissue were investigated (Fig 1). The diagnosis of cHL requires the microscopic evaluation of the tumor-engaged tissue[17]. Following primary diagnosis, patients are promptly treated[1]. It is therefore difficult to identify untreated patients with more than one biopsy with primary cHL. To address how PD-1 and PD-L1 varies in repeated biopsies in untreated patients with cHL, we reviewed the lymph node biopsies originally classified as non-malignant in patients that later developed cHL, with the intent that some non-malignant biopsies would be reclassified as cHL. Patients with at least two lymph node biopsies with cHL prior to start of treatment provided 11 paired cases. Patients with a biopsy at the primary diagnosis and a biopsy after primary treatment at relapse provided 30 paired cases (Fig 1).

### Treatment

Patients were treated according to national guidelines, which generally included chemotherapy with or without radiotherapy (Table 1). Since the material included patients diagnosed with primary cHL over a long time period (1980–2015), chemotherapy regimens differed (Table 1). Relapsed disease was treated with high-dose chemotherapy and autologous stem cell transplantation (ASCT) in young patients with limited comorbidity.

**Table 1:** Clinical characteristics of patients in the *treated* and the *untreated* group.

	Untreated group, n (%)	Treated group, n (%)
All patients	11 (100)	30 (100)
Age*		
≥45 years	5 (45)	11 (37)
<45 years	6 (55)	19 (63)
**Sex**		
Male	8 (73)	18 (60)
Female	3 (27)	12 (40)
**Histologic subtype**		
Nodular sclerosis	6 (55)	22 (73)
Mixed cellularity	5 (45)	8 (27)
Stage**		
IA-IIA	5 (45)	11 (37)
IIB-IVB	6 (55)	17 (57)
Missing	0 (0)	2 (6)
**Primary treatment received**		
Radiotherapy only	§	3 (10)
2–4 ABVD or MOPP/A(B)VD + RT	§	5 (17)
6–8 ABVD or MOPP/A(B)VD ± RT	§	10 (33)
6–8 BEACOPP	§	3 (10)
Other***	§	7 (23)
Missing	§	2 (7)
**Autologous stem cell transplantation at relapse**		
Yes	§	14
No	§	12
Missing	§	4

*Age at diagnosis of relapse in the treated group, and age at primary diagnosis in the untreated group.

**Stage at primary diagnosis.

***Other chemotherapy regimens were: OEPA (3 children and 1 elderly), one patient each received VACOP-B, Bendamustine and CHOP.

§Not applicable.

ABVD = Doxorubicine, Bleomycin, Vinblastine, Dacarbazine, BEACOPP = Bleomycin, Etoposide, Doxorubicine, Cyclophosphamide, Vincristine, Procarbazine, Prednisolone, CHOP = Cyclophosphamide, Hydroxydaunorubicin, Oncovin, Prednisolone, MOPP = Mustargen, Oncovin, Procarbazine, Prednisolone, OEPA = Oncovin, Etoposide, Prednisolone, Doxorubicine, VACOP-B = Etoposide, Doxorubicin, Cyclophosphamide, Vincristine, Prednisolone, Bleomycin

### Histological reclassification

Examination of the cases was performed by two of the authors. Of the 87 cases, 30 had insufficient FFPE material, and were excluded from the study. Twenty-nine cases had a lymph node biopsy classified as benign prior to a biopsy with cHL, and 11 (38%) of these were re-classified as cHL in biopsy 1 and confirmed to have cHL in biopsy 2. Since these patients received no treatment after biopsy 1, this group is referred to as the *untreated group*. Thirty paired samples with primary and relapsed cHL were confirmed during the diagnostic review. Since these patients received treatment after their primary diagnostic biopsy, this group is referred to as the *treated group*. Two patients were included in both the *untreated* and the *treated* group since they had tumor material available in biopsy 1 and biopsy 2, and also a biopsy of a subsequent relapse (Fig 1).

### Immunohistochemistry

As part of the diagnostic review, complementary immunohistochemical double-stainings for CD30 and paired box 5 protein (PAX5) were performed when needed. Cases were immunohistochemically stained with the Intellipath FLX automated staining system (Biocare Medical, Pacheco, CA). Single staining for PD-1 and double stainings for PD-L1/PAX5, and CD30/PAX5 were performed using 4 μm FFPE whole tumor sections. PD-1 was visualized with the mouse monoclonal antibody (mAb) NAT105/ab52587 (Abcam, Cambridge, UK), PD-L1 with the rabbit mAb E1L3N/13684 (Cell Signaling Technology, Danvers, MA), CD30 with the mouse mAb Ber-H2/IR602 (Dako, Santa Clara, CA). Two antibodies were used for PAX5 depending on whether it was double stained for PD-L1 or CD30, because the anti-PD-L1 was a rabbit antibody and the anti-CD30 was a mouse antibody. With PD-L1, PAX5 was visualized with the mouse mAb M7307/DAK-Pax5 (Dako), and with CD30, PAX5 was visualized with the rabbit polyclonal antibody ab140341 (Abcam). All antibodies were diluted 1:50. Antigen for PD-1 was retrieved in citrate buffer, and PD-L1, CD30 and both PAX5s were retrieved in TE buffer in a pressure cooker. PD-1 and CD30 were envisioned with MACH3 mouse HRP reagents (brown) (Biocare Medical), PD-L1 was envisioned with the Betazoid DAB detection kit (brown) (Biocare Medical), and both PAX5s were envisioned with the Warp Red chromogen (Biocare Medical). Finally, the sections were counterstained with Intellipath FLX´s hematoxylin.

### Evaluation of PD-1 and PD-L1

The proportions of PD-1+ and PD-L1+ leukocytes, and PD-L1+ HRS cells were manually quantified in 5 high power fields (HPF) at 400x (0.0625mm^2^). HPFs with visible HRS cells and most prevalent infiltration of PD-1+ or PD-L1+ cells were chosen and fibrosis was avoided. Cells with at least weak membranous staining for each marker were designated as positive, while other cells were designated as negative. The proportions were calculated by dividing the number of positive leukocytes by the total number of leukocytes (positive and negative), and the number of positive HRS cells by the total number of HRS cells. To investigate whether longer disease duration affected expression of PD-1 and PD-L1 in the *untreated* group, cases were grouped into short vs long duration between biopsy 1 and biopsy 2. The cut-off was set at the median time between biopsy 1 and biopsy 2, which was 5 months. To elucidate the prognostic impact regarding expression of PD-1 and PD-L1 in relapsed tumor samples, we applied cut-offs for the relapsed cases in the *treated group*, set at the 75^th^ percentile in order to divide patients into high vs low expression. High vs low proportion of PD-1+ leukocytes was defined as ≥5% vs <5% PD-1+ leukocytes. High vs low proportion of PD-L1+ leukocytes was defined as ≥46% vs <46% PD-L1+ leukocytes. High vs low proportion of PD-L1+ HRS cells was defined as ≥94% vs <94% PD-L1+ HRS cells.

### Epstein-Barr virus status

Biopsies with available FFPE material were stained with *in situ* hybridization technique to detect Epstein-Barr virus (EBV)-encoded small RNAs (EBER) in HRS cells in the *untreated group*.

### Statistical methods

The median was chosen to describe the average value for PD-1+ and PD-L1+ leukocytes, and PD-L1+ HRS cells, since we could not assume that the material was normally distributed. Wilcoxon signed-rank test was used to determine differences between continuous variables, i.e. proportions of PD-1+ and PD-L1+ leukocytes, and PD-L1+ HRS cells compared between biopsy 1 and biopsy 2 in the *untreated* group, and primary and relapse biopsy in the *treated* group. The survival outcome analyzed was time from relapse to death, defined as time from diagnosis of relapse confirmed by biopsy, to death from any cause. Survival differences between groups were calculated with Cox proportional hazards regression. A simple multivariate model included adjustment for age at relapse. Age was treated as a continuous variable. Statistics were analyzed with the R software and P values <0.05 were considered significant.

### Ethics

Ethical approval was obtained by the regional ethics committee in Uppsala, Sweden according to the declaration of Helsinki (2014/020, 2014/020/1, and 2014/233). Informed consent from participants was not required according to the ethics committee. All data were fully anonymized before accessed.

## Results

### Untreated group

In the *untreated* group, the median age was 43 years. Histologically, 6 (55%) cases had nodular sclerosis (NS) and 5 (45%) cases had mixed cellularity (MC) (Table 1).

#### Expression of PD-1 and PD-L1 in biopsy 1 and biopsy 2 in the untreated group

**Fig 2:**
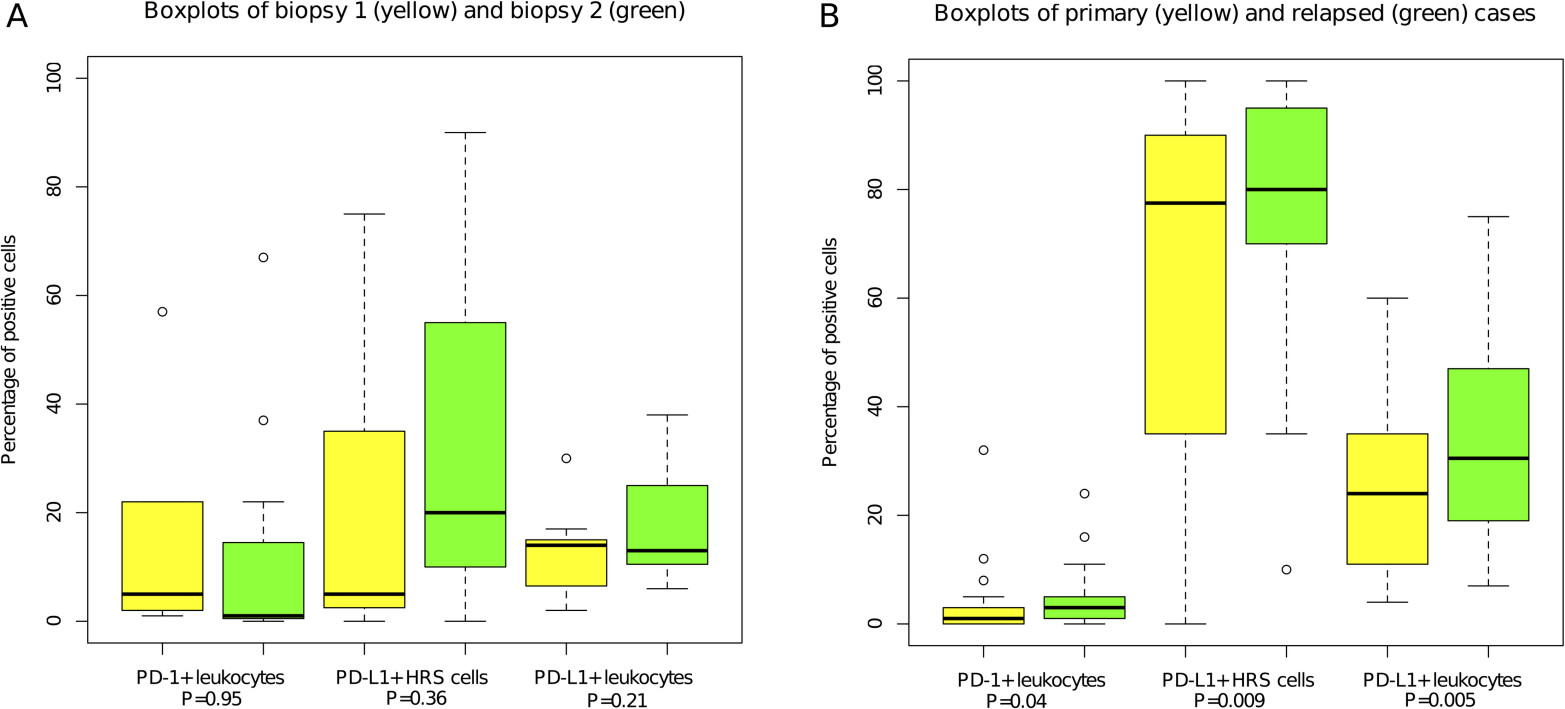
Boxplots of distributions of percentage of positive cells for (A) the *untreated* group and (B) the *treated* group. Wilcoxon signed-rank test P values are included comparing biopsy 1 and 2 in the *untreated* group, and primary and relapse biopsies in the *treated* group.

**Fig 3:**
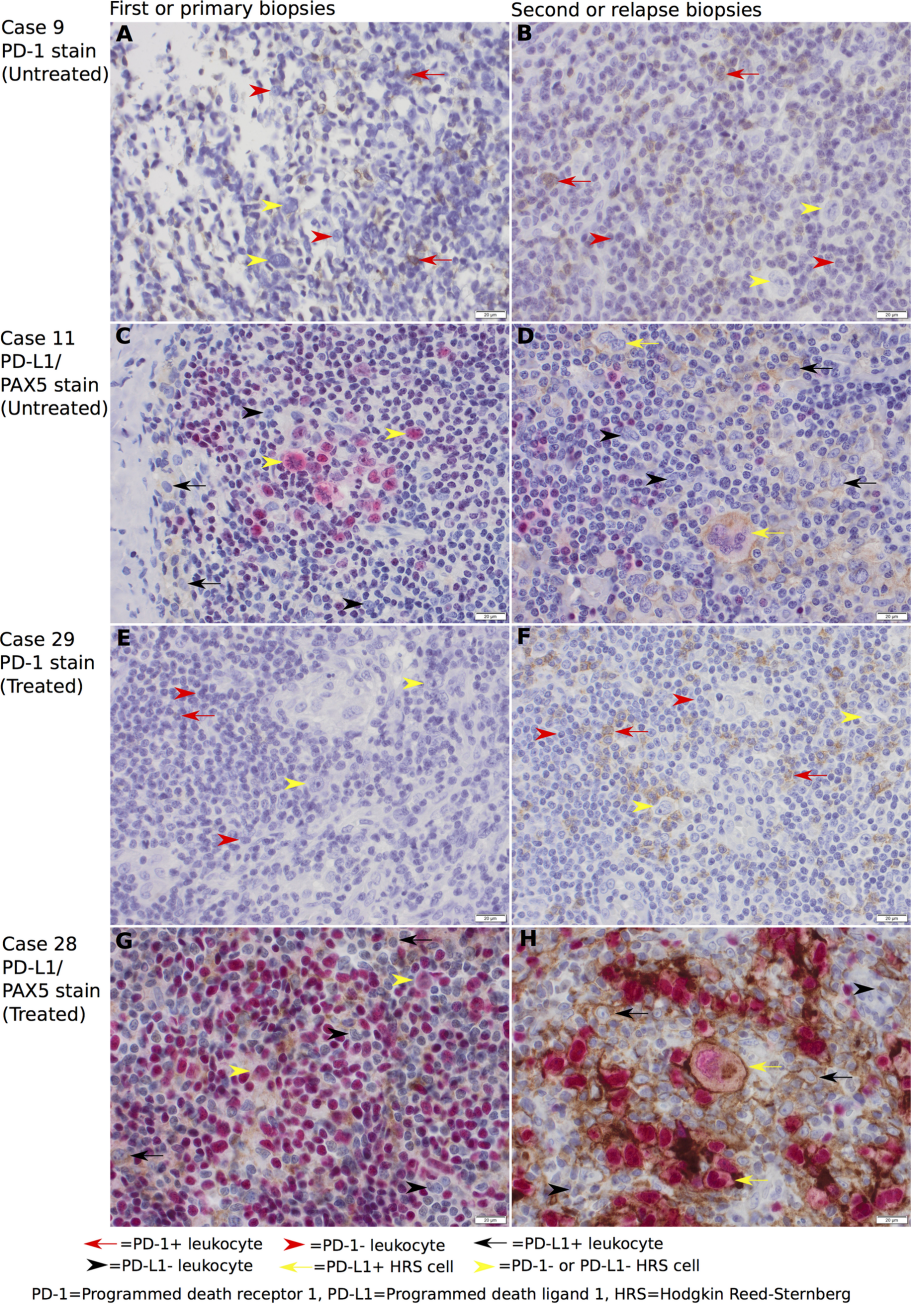
Representative PD-1 and PD-L1/PAX5 immunohistochemical stainings at 400x magnification with *untreated* (A-D) and *treated* (E-H) patients. Brown membranous staining indicates PD-1+ or PD-L1+ cells, while red nuclear staining indicates PAX5+ cells. *Untreated*: (A) Case 9 biopsy 1 (average 4% PD-1+ leukocytes), (B) case 9 biopsy 2 (average 7% PD-1+ leukocytes), (C) case 11 biopsy 1 (average 5% PD-L1+ leukocytes and 0% PD-L1+ HRS cells), and (D) case 11 biopsy 2 (average 10% PD-L1+ leukocytes and 100% PD-L1+ HRS cells). *Treated*: (E) Case 29 primary biopsy (average 1% PD-1+ leukocytes), (F) case 29 relapse biopsy (average 16% PD-1+ leukocytes), (G) case 28 primary biopsy (average 10% PD-L1+ leukocytes and 0% PD-L1+ HRS cells), and (H) case 28 relapse biopsy (average 19% PD-L1+ leukocytes and 100% PD-L1+ HRS cells).

For PD-1+ leukocytes, 4 (36%) cases had an increased expression, 5 (45%) a decreased expression, and 2 (18%) remained unchanged in biopsy 2 compared to biopsy 1 (Fig 2A), median proportion was 5% in biopsy 1 and 1% in biopsy 2. For PD-L1+ leukocytes, 8 (73%) had an increased and 3 (27%) had a decreased expression in biopsy 2 compared to biopsy 1, median proportion was 14% in biopsy 1 and 13% in biopsy 2 (Fig 2A). For PD-L1+ HRS cells, 6 (55%) cases had an increased expression, 2 (18%) cases a decreased expression, and 3 (27%) remained unchanged in biopsy 2 compared to biopsy 1, median proportion was 5% in biopsy 1 and 20% in biopsy 2 (Fig 2A). With Wilcoxon signed-rank test, there were no statistically significant differences considering the median difference between biopsy 1 and biopsy 2 considering PD-1+ leukocytes (p = 0.95), PD-L1+ leukocytes (p = 0.21), or PD-L1+ HRS cells (p = 0.36). Examples of immunohistochemically stained slides with PD-1 for case 9 (Fig 3A and 3B), and with PD-L1/PAX5 for case 11 (Fig 3C and 3D) are shown in Fig 3.

#### Differences in expression of PD-1 and PD-L1 with disease duration in the untreated group

In most cases, less than a year elapsed between biopsy 1 and biopsy 2 (median 5 months). However, case 11 showed an exceptionally long time (66 months) between the biopsies, with a marked elevation in the proportion of PD-L1+ HRS cells, from 0% in biopsy 1 to 80% in biopsy 2. All cases that expressed PD-L1 in the HRS cells in biopsy 1 also expressed PD-L1 in the HRS cells in biopsy 2 (Table 2).

**Table 2:** Clinicopathological characteristics and proportion of PD-1+ leukocytes, PD-L1+ leukocytes and PD-L1+ HRS cells in the untreated group.

Case	Calendar year^a^	Time between biopsies (months)^b^	Age^c^	EBV status	Location^d^	Original diagnosis^e^	Histology^f^	PD-1+ leukocytes (%)		PD-L1+ leukocytes (%)		PD-L1 +HRS (%)	
								Biopsy 1	Biopsy 2	Biopsy 1	Biopsy 2	Biopsy 1	Biopsy 2
1	1991	1	43	Neg	Inguinal	Suspected lymphoma	MC	57	67	30	9	5	20
2	1986	1	75	Pos	Neck	Infectious disease	MC	22	0	14	11	5	10
3	1990	2	42	Pos	Abdomen	Suspected lymphoma	MC	7	1	2	11	0	0
4	1993	2	33	Neg	NA	Suspected lymphoma	NS	22	37	8	25	0	0
5	2010	4	6	Neg	Axilla	Infectious disease	NS	2	1	15	33	5	20
6	1991	5	24	Pos	Inguinal	Reactive lymphadenopathy	NS	1	0	15	25	35	90
7	1997	7	32	Neg	Neck	Pathologic, no malignancy	NS	5	7	5	13	50	70
8	2001	8	66	Neg	Neck	Pathologic, no malignancy	MC	1	1	10	13	10	10
9	2013	8	47	Neg	Abdomen	Suspected lymphoma	NS	4	7	15	38	75	40
10	1998	13	66	Neg	Inguinal	Reactive lymphadenopathy	MC	22	22	17	6	35	10
11	2000	66	63	Neg	Abdomen	Reactive lymphadenopathy	NS	2	0	5	10	0	80

^a^Year of initial diagnosis of cHL.

^b^ Time elapsed between biopsy 1 and biopsy 2 in months.

^c^Age at initial diagnosis of cHL.

^d^Biopsy 1 and biopsy 2 were taken from the same location in all cases.

^e^At biopsy 1.

^f^At biopsy 2.

PD-1 = Programmed death receptor 1, PD-L1 = Programmed death ligand 1, HRS = Hodgkin Reed-Sternberg, MC = Mixed cellularity, NS = Nodular sclerosis, EBV = Epstein-Barr virus, NA = Not available.

#### EBV is constantly expressed in the HRS cells in biopsy 1 and biopsy 2 in the untreated group

Three of 11 cases (27%) were EBV positive in the HRS cells in both biopsy 1 and 2 (Table 2). If biopsy 1 was negative or positive, the corresponding biopsy 2 was also negative or positive, respectively.

### Treated group

In the *treated* group, the median age at primary diagnosis was 32.5 years, the median age at relapse was 36.5 years and the median time to relapse was 3 years. Histologically, 73% had NS and 27% had MC at primary diagnosis (Table 1). One had NS histology at primary diagnosis and MC at relapse, while all others had the same histology at relapse as in the primary biopsy. Following relapse, 8 (27%) patients died and median follow-up was 6.5 years (range 0.3–30.9 years).

#### PD-1 and PD-L1 are upregulated in the relapse biopsies compared to the primary biopsies in the treated group

More cases showed an upregulation rather than a downregulation in the relapse biopsy compared to the primary biopsy for all markers (Fig 2B). For PD-1+ leukocytes, 19 cases (63%) showed a proportional increase, 7 (23%) a decreased expression, and 4 (13%) remained unchanged in the relapse biopsy compared to the primary biopsy, median proportion was 1% in the primary biopsy and 3% in the relapse biopsy (Fig 2B). For PD-L1+ leukocytes, 22 cases (73%) had an increased expression, 7 (23%) a decreased expression, and 1 (3%) remained unchanged in the relapse biopsy compared to the primary biopsy, median proportion was 24% in the primary biopsy and 30.5% in the relapse biopsy (Fig 2B). For PD-L1+ HRS cells, 18 cases (60%) had an increased expression, 3 (10%) a decreased expression, and 9 (30%) remained unchanged in the relapse biopsy compared to the primary biopsy, median proportion was 77.5% in the primary biopsy and 80% in the relapse biopsy (Fig 2B). The increases between the primary and relapse biopsies were statistically significantly different considering PD-1+ leukocytes (p = 0.04), PD-L1+ leukocytes (p = 0.005), and PD-L1+ HRS cells (p = 0.009) (Fig 2B). In addition, we observed that all 30 (100%) relapse biopsies expressed PD-L1 on the HRS cells to some degree (>0%), while it was expressed to some degree in 26 (87%) of the primary biopsies (Table 3). Examples of immunohistochemically stained slides with PD-1 for case 29 (Fig 3E and 3F) and with PD-L1/PAX5 for case 28 (Fig 3G and 3H) are shown in Fig 3.

**Table 3:** Clinicopathological characteristics and proportion of PD-1+ leukocytes, PD-L1+ leukocytes and PD-L1+ HRS cells in the treated group*.

Case	Calendar year^a^	Time between biopsies^b^	Age^c^	EBV status^d^	Biopsy location (primary/relapse)	Diagnosis (primary/relapse)	PD-1+ leukocytes (%)		PD-L1+ leukocytes (%)		PD-L1 +HRS (%)	
							Primary	Relapse	Primary	Relapse	Primary	Relapse
1	2005	20	26	NA	abdomen/abdomen	MC/MC	1	3	19	19	100	100
2	2006	112	41	NA	neck/neck	NS/NS	0	1	27	40	100	100
3	2010	40	35	NA	neck/mediastinum	NS/NS	0	3	37	75	100	100
4	2010	76	12	NA	neck/mediastinum	MC/MC	0	8	41	48	100	70
5	2015	8	26	negative	neck/neck	NS/NS	5	4	45	57	100	100
6	1997	31	31	NA	neck/neck	NS/NS	0	1	40	31	95	100
7	2001	23	37	NA	neck/neck	NS/NS	0	2	16	20	90	90
8	1991	17	24	NA	inguinal/NA	NS/NS	0	1	25	30	90	70
9	2000	10	16	NA	neck/neck	NS/NS	1	1	31	29	90	90
10	2009	41	25	NA	neck/neck	NS/NS	0	3	34	50	90	95
11	2015	7	32	negative	neck/neck	NS/NS	3	5	38	12	90	90
12	1998	45	46	NA	neck/neck	NS/MC	0	3	60	43	85	50
13	2003	36	45	NA	neck/neck	NS/NS	1	0	26	29	80	80
14	1984	136	32	NA	neck/paraaortal	MC/MC	2	1	33	34	80	80
15	2007	15	55	NA	inguinal/inguinal	MC/MC	1	1	35	43	80	85
16	1980	146	36	negative	neck/neck	NS/NS	3	11	22	53	75	90
17	1981	72	27	NA	neck/neck	NS/NS	1	3	4	17	70	90
18	2009	8	65	NA	axilla/NA	NS/NS	0	1	21	26	65	75
19	2014	12	16	NA	axilla/inguinal	NS/NS	3	7	25	58	60	70
20	2011	8	33	NA	neck/neck	NS/NS	4	1	10	37	50	80
21	1981	64	16	NA	NA/NA	MC/MC	0	0	18	47	45	100
22	1989	43	75	NA	inguinal/inguinal	NS/NS	3	0	10	18	40	70
23	2014	17	64	negative	inguinal/inguinal	NS/NS	32	8	18	20	35	70
24	2005	7	76	positive	neck/neck	MC/MC	0	1	46	34	30	35
25	1986	36	29	NA	mediastinum/neck	NS/NS	8	24	8	17	15	40
26	1994	49	13	NA	neck/lung	NS/NS	12	2	8	7	10	40
27	1989	20	12	NA	axilla/axilla	NS/NS	0	5	4	11	0	80
28	2011	68	68	NA	neck/neck	NS/NS	4	6	10	19	0	100
29	1990	53	42	NA	abdomen/NA	MC/MC	1	16	11	9	0	10
30	1981	48	39	NA	NA/NA	MC/MC	3	3	23	73	0	80

^a^Year of primary diagnosis of cHL.

^b^Time elapsed between biopsy 1 and biopsy 2 in months.

^c^Age at primary diagnosis of cHL.

^d^EBV status determined with EBER at primary biopsy.

PD-1 = Programmed death receptor 1, PD-L1 = Programmed death ligand 1, HRS = Hodgkin Reed-Sternberg, MC = Mixed cellularity, NS = Nodular sclerosis, EBV = Epstein-Barr virus, NA = Not available.

*Sorted according to expression of PD-L1 by HRS cells.

#### PD-1 and PD-L1 in the relapse biopsy in relation to prognosis in the treated group

High proportions of PD-1+ and PD-L1+ leukocytes, and PD-L1+ HRS cells did not affect time to death after relapse in univariate or age-adjusted analyses. The only prognostic discriminator was age at relapse, which was associated with a shorter time to death after relapse, hazard ratio: 1.05 (95% confidence interval, 1.002–1.09, p = 0.04).

## Discussion

**Fig 4:**
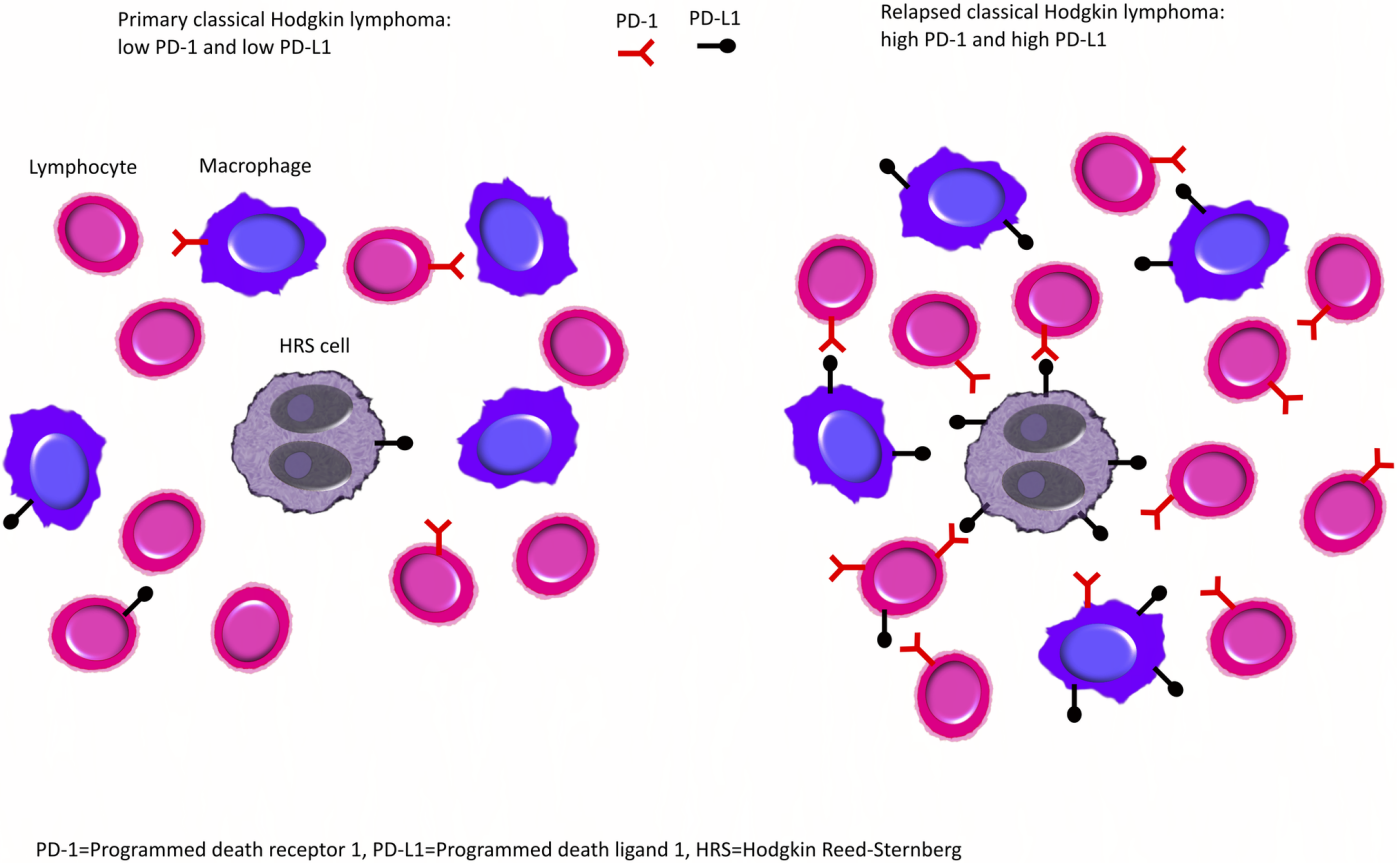
Visual summary of the main findings in the study.

This study describes the biologic variation over time of the PD-1 pathway in cHL. In paired biopsies from previously *untreated* and previously *treated* patients with primary and relapsed cHL, respectively, we found that PD-1 and PD-L1 were upregulated in the relapsed (*treated)* group, and a tendency that PD-L1+ leukocytes were upregulated in the *untreated* group (Fig 4).

### Possible clinical applications

Upregulation of the PD-1 pathway is primarily of biologic interest, but could also be relevant when PD-1 blockade is tested as front-line treatment[18]. PD-1 blockade is only currently routinely used in refractory and relapsed cHL, with high treatment response rates[19], particularly in those with high expression of PD-L1 on HRS cells[20, 21]. In other malignancies, conventional chemotherapy upregulates expression of PD-L1 on tumor cells in cases of primary head and neck cancer[22] and lung adenocarcinoma[15]. It is unknown whether conventional front-line chemotherapy and radiotherapy upregulates expression of PD-L1 on HRS cells in cHL, however our findings with a higher expression of PD-L1 in relapsed than in primary biopsies indicate such an association. In chronic lymphocytic leukemia patients with Richter transformation, high overall expression of PD-1 and PD-L1, and prior treatment with ibrutinib resulted in a more favorable response to PD-1 inhibition[23]. The most suitable chemotherapy and radiotherapy in combination with PD-1 inhibition, and optimal sequences between chemotherapy and radiotherapy, and PD-1 inhibition should be further explored in clinical trials.

### Expression of PD-1 and PD-L1 in the treated and the untreated group

Most (60–73%) patients in the *treated* group had a higher expression of each marker in their relapse biopsy compared to their primary biopsy, demonstrating that the PD-1 pathway appears to be upregulated at relapse in cHL. We also found that all relapse biopsies expressed PD-L1 in the HRS cells at least to some degree, which is compatible with previous studies where PD-L1 was expressed by HRS cells in all relapsed cases with cHL[7, 8]. The findings were less convincing for the *untreated* group; nevertheless 73% of the patients had an increased proportion of PD-L1+ leukocytes in biopsy 2 compared to biopsy 1, indicating that also disease progression itself, and not only treatment might upregulate PD-L1 expression in leukocytes. EBV was present in the HRS cells in 3 cases in the *untreated* group, but there was no change in EBV status when biopsy 1 and biopsy 2 were compared.

### Mechanism for upregulation of PD-L1 on HRS cells

An upregulation of PD-L1 on HRS cells at relapse might include several mechanisms, mainly genetic alterations following anti-cancer treatment, but may also reflect longer disease duration. As mentioned previously, how contemporary front-line chemotherapy regimens alter the PD-1 pathway in cHL has never been investigated earlier. However, treatment has been associated with an increased expression of PD-L1 on tumor cells in other malignancies. In a mouse breast cancer model, radiotherapy upregulated expression of PD-L1 on tumor cells[24]. In a colorectal cancer cell line, 5-fluorouacil upregulated expression of PD-L1 on tumor cells[25] in primary tumors. HRS cells frequently harbor copy gains in the gene 9p24.1, which is associated with expression of the PD-1 ligands on the HRS cells[26]. It is known that chemotherapy might affect gene status in NSCLC[27]. Primary treatment might induce genetic alterations in the 9p24.1 gene, which could explain the higher proportion of PD-L1+ HRS cells at relapse.

### Mechanism for upregulation of PD-L1 on leukocytes

cHL is characterized by an extensive inflammatory infiltrate consisting of various leukocytes[28]. In malignant diseases, PD-1 ligands become upregulated on leukocytes in the tumor microenvironment by various pro-inflammatory cytokines, e.g. interferon γ, interleukin 10, and tumor necrosis factor α, produced by tumor cells and other leukocytes such as T cells[3, 29–31]. Thus, a more pro-inflammatory tumor milieu is a plausible explanation for our findings, i.e. a higher proportion of PD-L1+ leukocytes in the microenvironment in relapse biopsies in the *treated* group, and an indication of a higher proportion of PD-L1+ leukocytes in the *untreated* group. In addition, in the mouse breast cancer model referred to above, radiotherapy upregulated expression of PD-L1 on dendritic cells[24]. Thus, treatment also likely induces a higher proportion of PD-L1+ leukocytes, as we noted in the *treated* group.

### Mechanism for upregulation of PD-1 on leukocytes

PD-1 is expressed by different activated leukocytes (e.g. lymphocytes, dendritic cells, and macrophages), and is upregulated on T cells following persistent exposure to antigens[3, 32]. This was also seen in the study on the colorectal cancer mouse model[13], where PD-1+ macrophages were upregulated following longer disease duration without treatment. In addition, patients with relapsed multiple myeloma had a more pronounced infiltration of PD-1+ lymphocytes after than before treatment[16]. Increased proportions of PD-1+ leukocytes in relapsed cases in the *treated* group might thus be a result of persistent or previous exposure to antigens.

### Altered expression of PD-1 and PD-L1 related to treatment or time?

To summarize the previous paragraphs: upregulation of PD-1 and PD-L1 can be explained by a more pronounced inflammatory microenvironment following longer disease duration, changed microenvironmental milieu in the relapsed setting, or the primary treatment. The median time between the primary and the relapse biopsy in the treated group was considerably longer than the median time between biopsy 1 and biopsy 2 in the untreated group, 36 and 5 months, respectively. Thus, it is difficult to determine whether it is the treatment, disease progression, altered conditions in the relapsed microenvironment, or all combined that are most important for the upregulation of PD-1 and PD-L1.

### PD-1 and PD-L1 importance for survival in relapsed cases

In a previous study, we found that high proportions of PD-1+ and PD-L1+ leukocytes in primary tumors with cHL were associated with inferior outcome, while expression of PD-L1 on HRS cells had no impact on outcome[9]. In the current study, no association with survival according to high vs low expression of PD-1+ and PD-L1+ leukocytes, and PD-L1+ HRS cells was found. However, only 27% of the patients in our cohort died, thus a considerably lower proportion in comparison with a large retrospective Swedish cohort where approximately 50% of the patients died following relapse[1]. Re-biopsied patients are probably more prone to receive treatment with curative intent, than patients for whom a new biopsy of a suspected relapse is not performed. Thus, our material likely favored to mainly include young and healthy individuals with little comorbidity.

### Methodological considerations for immunohistochemical stainings

Both lymphocytes and antigen presenting cells (i.e. macrophages, monocytes and dendritic cells) are able to express both PD-1 and PD-L1 in malignancies[32] Since it is troublesome to differentiate large lymphocytes from small macrophages and monocytes based solely on morphologic examination, we decided to adapt a summary measure and term them as leukocytes. However, it is known that PD-1 is mostly expressed by T-cells, and PD-L1 is mostly expressed by macrophages in cHL[29]. This seemed true in our material in general when manually evaluated, which is also exemplified in Fig 3 where PD-1 is mainly expressed by smaller lymphocytes, and PD-L1 is mainly expressed by larger macrophages. Hence, our PD-1 findings are mostly based on expression by lymphocytes while PD-L1 findings are mostly based on expression by macrophages.

### Methodological considerations for the untreated group

Core needle biopsies may contain insufficient material to diagnose lymphomas[33]. However, for the *untreated* group, only biopsy 1 for case 3 and 4, and biopsy 2 for case 10 (Table 2) were core needle biopsies, while the rest were whole lymph node excisions. Hence, in most cases the original misclassification is probably not explained by inadequate material provided in biopsy 1. HRS cells have a distinct immunohistochemical profile, being positive for CD30 and PAX5[17]. For biopsy 1, only 2 cases were examined with immunohistochemical markers in the original diagnostic process. Most of the cases not examined with immunohistochemistry were diagnosed prior to the year 2000 (Table 2), when immunohistochemistry was less commonly used. We reclassified 38% of the first biopsies as cHL, likely explained by our utilization of immunohistochemical markers in combination with a confirmatory biopsy with cHL in biopsy 2, which simplified our review procedure compared to the original diagnostic procedure.

### Strengths and limitations

To our knowledge, no previous publication has investigated the expression of PD-1 and PD-L1 in repeated biopsies in patients with cHL. However, due to treatment recommendations advocating immediate treatment in HL, only 11 cases had consecutive biopsies where patients remained untreated, despite screening 3500 cases. This limited the statistical power and our results should be viewed as hypothesis generating. For all patients in the *untreated* group, both biopsy 1 and biopsy 2 were taken from the same location, minimizing the bias of possibly different composition of the microenvironment in the same patient between different localizations. The unique material and design of our study make an important knowledge contribution regarding immune checkpoint regulation in cHL.

### Conclusions

This is the first study to describe that PD-1 and PD-L1 are upregulated in relapsed cHL compared to primary diagnosis, and the tendency that PD-L1+ leukocytes are upregulated with time in untreated cHL. This further adds knowledge about the complex inflammatory infiltrate orchestrated by the PD-1 pathway in cHL. Future studies are warranted to elucidate whether it is longer disease duration, primary treatment, or altered conditions in the relapse microenvironment that upregulate the PD-1 pathway. However, based on numerous previous studies[15, 16, 23–25], PD-1 and PD-L1 are most likely upregulated due to previous treatment with chemotherapy and radiotherapy, which could have implications regarding treatment with PD-1 inhibition in cHL.

## Supporting information

S1 Table Untreated group. (XLSX)

S2 Table Treated group. (XLSX)
